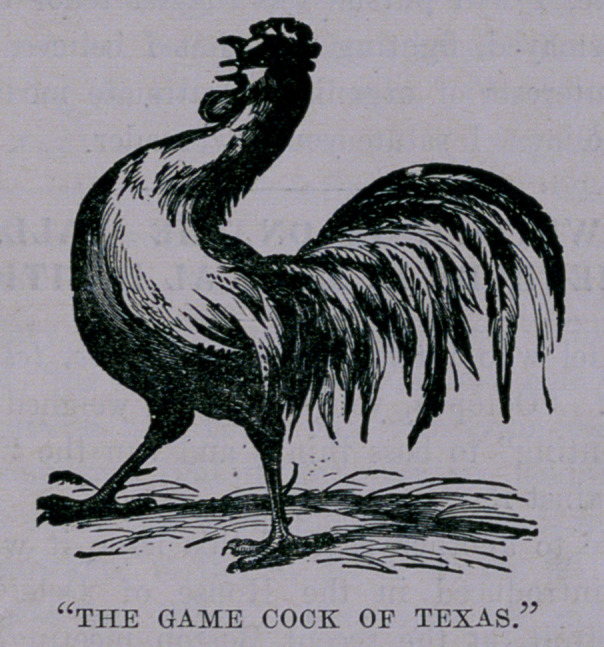# Our Anniversary

**Published:** 1906-07

**Authors:** 


### EDITORIAL DEPARTMENT.

"THE GAME COCK OF TEXAS."

### OUR ANNIVERSARY.

The *Red Back* is 21 years old today, July 10. It has been twenty-one years of SUCCESS—of strenuosity, of warfare against quackery, and of advocacy and defense of legitimate medicine. Twenty-one years ago the JOURNAL spread its sails and hoisted this banner:

**In Hoc Signo Vinces:**

**"Independent in all things and neutral in nothing that affects the welfare of Legitimate Medicine. It is devoted to the task of organizing the Texas profession for its own safety and protection, and to acquire influence in shaping the Sanitary Laws of the State; to the advancement of Medical Science and the elevation of the Standard of Medical Education. IT IS DOWN ON QUACKS OF ALL KINDS."**

It has been a sensation and an inspiration. When I reflect upon the favor with which it has always been received, and the cordial, enthusiastic support accorded it, and the good work it has accomplished, and the further fact that with age it has not only retained the favor and support of the very best element of the profession, but has grown in favor and influence until it is a power, I feel a little disposed to crow, and am quite excusable for crowing. I feel like celebrating.

I make my bow to my army of supporters, and from my heart thank them for their loyalty. And I feel like I am good for another twenty-one years. So here goes for the twenty-second round, with a smile for those who love me and a cuss word for those who hate, I will pursue the rugged tenor of my way, undaunted, undismayed, fighting for what I believe to be *right* and for the best interests of organized legitimate medicine; for principle versus policy. I salute you, my reader.

## Figures and Tables

**Figure f1:**